# Inter-individual differences in contamination profiles as tracer of social group association in stranded sperm whales

**DOI:** 10.1038/s41598-018-29186-z

**Published:** 2018-07-19

**Authors:** Joseph G. Schnitzler, Marianna Pinzone, Marijke Autenrieth, Abbo van Neer, Lonneke L. IJsseldijk, Jonathan L. Barber, Rob Deaville, Paul Jepson, Andrew Brownlow, Tobias Schaffeld, Jean-Pierre Thomé, Ralph Tiedemann, Krishna Das, Ursula Siebert

**Affiliations:** 1 Institute for Terrestrial and Aquatic Wildlife Research, University of Veterinary Medicine Hannover, Foundation, 25761 Büsum, Schleswig-Holstein Germany; 2 Freshwater and Oceanic sciences Unit of reSearch - Oceanology, University of Liège, Allée du 6 Août, B6C, 4000 Liège, Belgium; 3 Unit of Evolutionary Biology/Systematic Zoology, Institute for Biochemistry and Biology, University of Potsdam, Karl-Liebknecht-Str. 24-25, 14476 Potsdam, Germany; 4 Faculty of Veterinary Medicine, Department of Pathobiology, Utrecht University, Yalelaan 1, 3584CL Utrecht, The Netherlands; 5 Centre for the Environment, Fisheries and Aquaculture Science (Cefas) Lowestoft Laboratory, Pakefield Road, Lowestoft, Suffolk NR33 0HT UK; 6 CSIP, Institute of Zoology, Regent’s Park, London, NW1 4RY UK; 7 SRUC Wildlife Unit, Drummondhill, Inverness, IV2 4JZ UK; 8 Laboratory of Animal Ecology and Ecotoxicology (CART-LEAE) B6c, Liège University, Liège, Belgium

## Abstract

Ecological and physiological factors lead to different contamination patterns in individual marine mammals. The objective of the present study was to assess whether variations in contamination profiles are indicative of social structures of young male sperm whales as they might reflect a variation in feeding preferences and/or in utilized feeding grounds. We used a total of 61 variables associated with organic compounds and trace element concentrations measured in muscle, liver, kidney and blubber gained from 24 sperm whales that stranded in the North Sea in January and February 2016. Combining contaminant and genetic data, there is evidence for at least two cohorts with different origin among these stranded sperm whales; one from the Canary Island region and one from the northern part of the Atlantic. While genetic data unravel relatedness and kinship, contamination data integrate over areas, where animals occured during their lifetime. Especially in long-lived animals with a large migratory potential, as sperm whales, contamination data may carry highly relevant information about aggregation through time and space.

## Introduction

Sperm whales (*Physeter macrocephalus*), the largest toothed whale (Odontoceti), are among the most social of the great whales. Except during breeding seasons, adult male and female sperm whales are geographically segregated. The group structure, sizes, home ranges and codas of female groups are relatively well studied^1^, whereas little is known about the group structure of male sperm whales.

Adult females live in cohesive groups along with juveniles of both sexes in primarily low-latitude waters of tropical and subtropical waters. The sub-adult males disperse from their natal unit at an approximate age of 10 years and tend to move gradually to higher latitudes into colder surface waters^2–4^. These young sperm whales are regularly found in all-male bachelor groups which are believed to be composed of constant companions and casual acquaintances^2,5–8^. Aside from these bachelor groups, male sperm whales are usually seen solitary or in occasional pairs of sexually and physically mature males as they age^2,3^ and appear to roam over large distances^2,3,9^. In their late twenties, the sexually mature males eventually return to lower latitudes to the breeding grounds inhabited by females to mate^2,9–11^.

Especially unexplored are the size and structure of bachelor groups which are believed to be loose aggregations of similar-sized males that have left their mother’s social units. Organic compound and trace elements concentrations could be used as a tool for identifying their affiliation to social groups within the species^12–14^. It is known that ecological, biological and physiological factors lead to different contamination patterns in individual marine mammals^12–16^. With regards to male sperm whales, which are generally good sentinels of ecosystem health as they continuously accumulate pollutants, the habitat where these animals feed is likely to be a factor that strongly influences organic compound and trace element concentration profiles. Each congener or element has its own trophic source, and persistent organic pollutant (POP) or trace element (TE) profiles have been used as a fingerprint to infer dietary habits of toothed whales^17–19^. High pollutant burdens are generally found in coastal regions due to the close proximity to possible emissions, discharges and losses of pollutants in temperate areas^20,21^. The pattern of Organohalogen Compounds from the same chemical group, such as polychlorinated biphenyls (PCBs) and polybrominated diphenyl ethers (PBDEs) may differ according to the distance from the source^22–24^. The proportion of the highly halogenated congeners decreases with distance from the source, as the lighter congeners are more volatile and are capable of being transported over longer distances^25^. Trace elements are released into the environment from both natural (e.g., volcanism) and anthropogenic (e.g., industrial, urban, or agricultural) sources and may thus show local specificities due to river inputs and atmospheric depositions^26^. Therefore, a variation in prey preference and/or feeding location will result in varying tissue concentrations and patterns of organic compound and trace elements among different individuals of the same whale species^19^.

In this context, the objective of the present study was to assess whether variations of contamination profiles are indicative of social structures of young male sperm. The second objective was to assess which contaminants can be discriminated efficiently between individuals and thus have the potential to be used as ecological tracers. Additionally, these findings will be analysed in the context of genetic relationship and kinship. To meet these objectives, we used a total of 61 variables associated with organic compound and trace element concentrations measured in muscle, liver, kidney and/or blubber from 24 sperm whales that stranded in the North Sea in January and February 2016.

## Results

**Figure 1:**
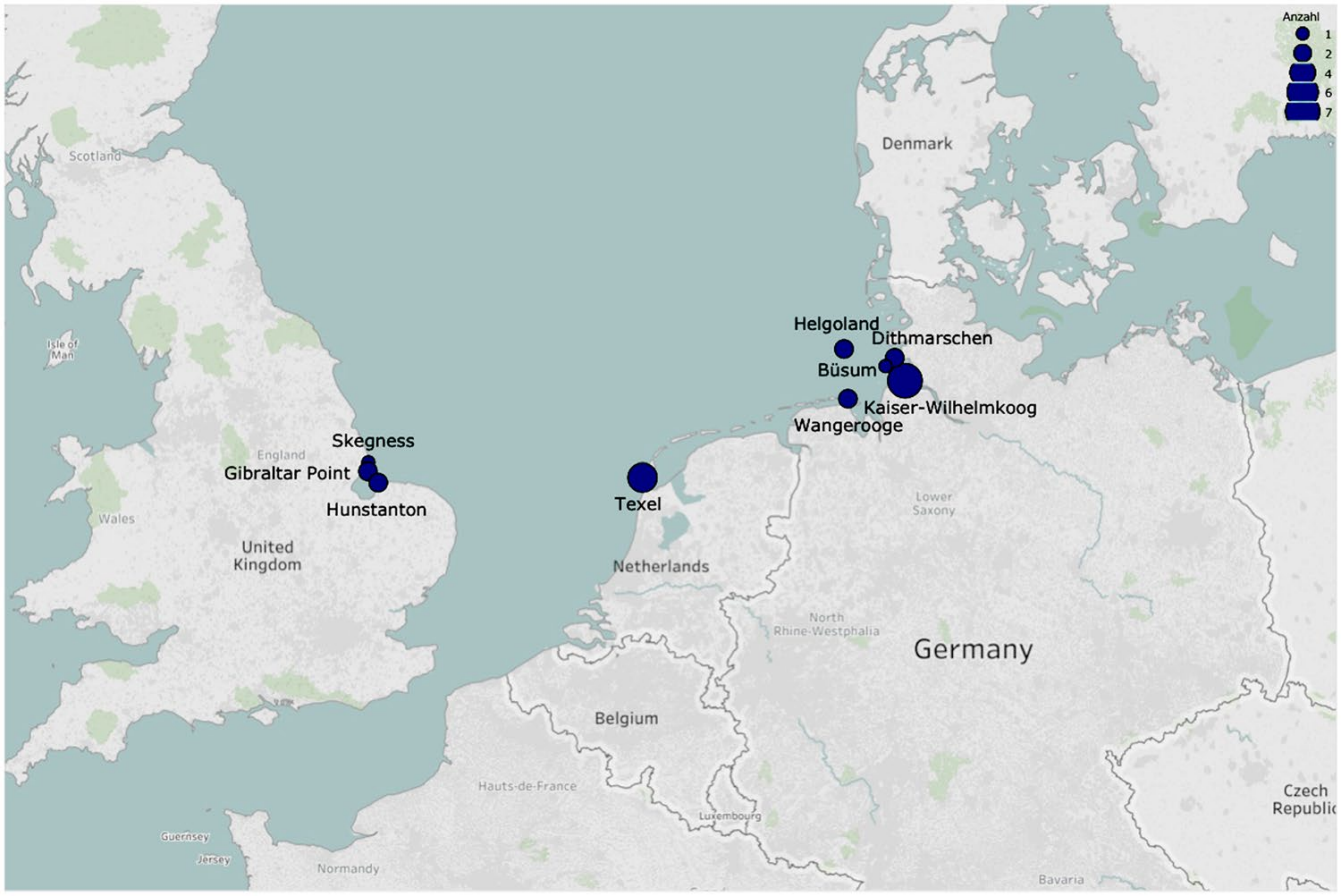
Sperm whale stranding sites (Jan–Feb 2016) visualized on a map using Tableau version 9.3 with Tableau base-map country outlines. Size of circle equals number of individuals necropsied at that site.

**Figure 2:**
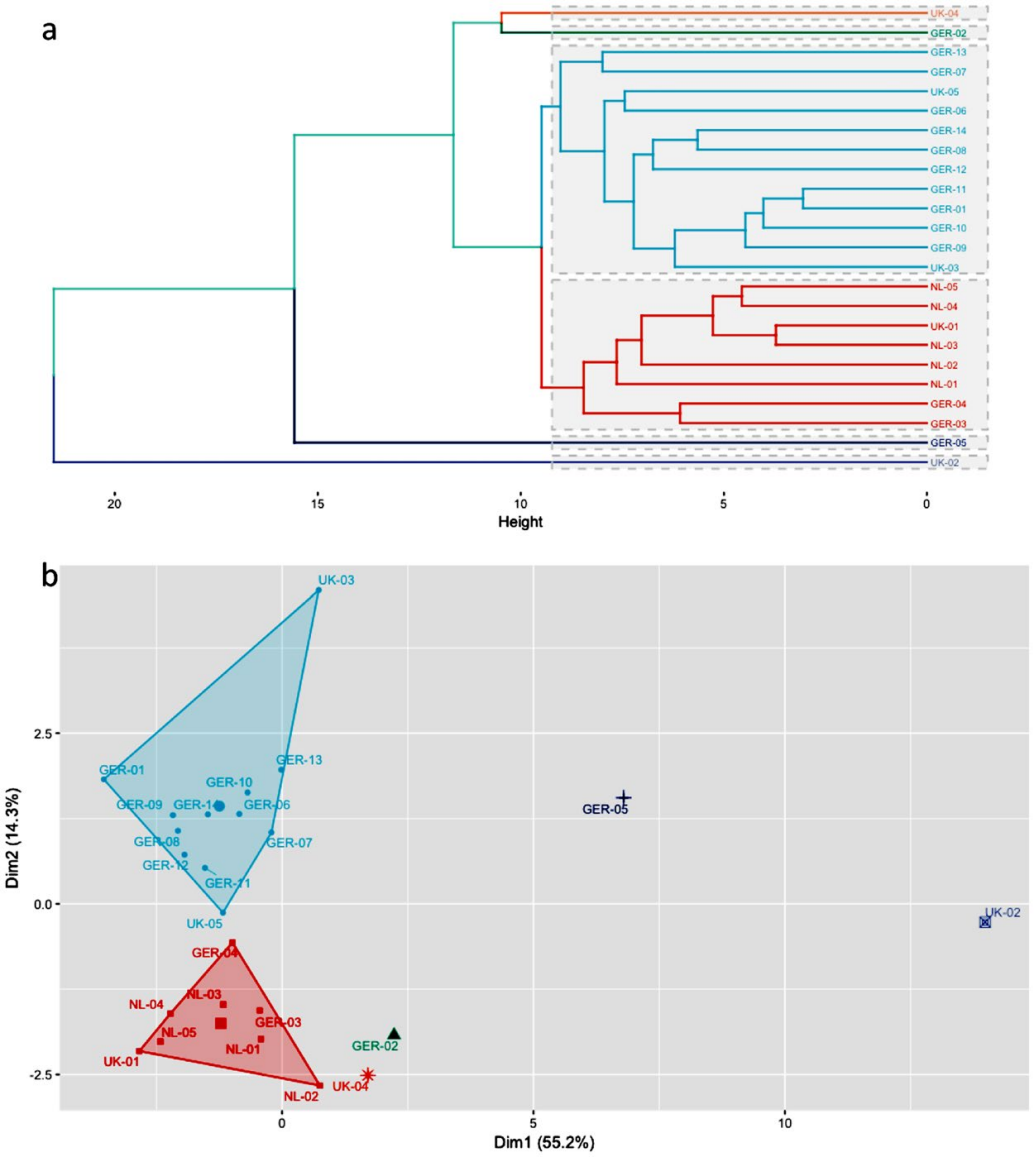
Discrimination of the 24 stranded sperm whales: (**a**) Distance phenogram summarizing the UPGMA clustering of 24 stranded sperm whales based on 61 variables associated with organic compound and element concentrations in either muscle, liver, kidney or blubber. The cophenetic correlation is 0.89. (**b**) Projections of the 24 stranded sperm whales onto the first two principal components based on 61 variables associated with organic compound and element concentrations in either muscle, liver, kidney or blubber. The cluster 1 (January) is marked in red, the cluster 2 (February) in light blue and the 4 solitaires are marked respectively in orange, green, dark blue and violet.

In a period of five weeks, thirty sperm whales stranded^27^ of which twenty-four were necropsied on the North Sea shores (Fig. 1). All sperm whales were young males aged between 10 and 15 years and had an average total body length of 11.7 m, ranging between 9.6–14.7 m (Table 1). A total of 61 variables associated with organic compound and element concentrations in muscle, liver, kidney and blubber were analysed when possible for all 24 necropsied sperm whales in order to characterize their contamination profile. We performed a hierarchical cluster analysis and Principal Component Analysis (PCA) based on these traits to reveal similarities of contamination profiles among individuals (Fig. 2a). The correlation between the original distances and the cophenetic distances was high (coefficient = 0.89), indicating that the dendrogram summarises the data appropriately. The unweighted pair-group method with arithmetic average algorithm (UPGMA) separates two main clusters: (1) a group of eight sperm whales that stranded all in January and (2) a second group including predominantly the sperm whales that stranded in early February.

**Table 1:** Basic biology data gathered from stranded sperm whales: country, date of first report (dd/mm/yyyy), stranding location, coordinates (latitude & longitude), age (y), length (m) and *measured weight or estimated weight (Weight (T) = 0.006648 Length^3.18^;^62^.

Number	Country	Date	Location	Latitude	Longitude	Age (y)	Total length (m)	Weight (T)
GER01	Germany	08/01/2016	Wangerooge	53°78′05.78″N	7°97′56.6″E	nd	11.8	17.0
GER02	Germany	08/01/2016	Wangerooge	53°78′05.78″N	7°97′56.6″E	nd	13.10	23.7
GER03	Germany	12/01/2016	Helgoland	54°21′46.1″N	7°91′31.18″E	13	12.0	18.0
GER04	Germany	12/01/2016	Helgoland	54°19′15.9″N	7°89′19.93″E	13	12.3	19.4
NL01	Netherlands	12/01/2016	Texel	54°08′51.79″N	8°58′88.61″E	nd	9.6	8.8
NL02	Netherlands	12/01/2016	Texel	53°03′85.62″N	4°71′19.65″E	nd	11.1	14.0
NL03	Netherlands	12/01/2016	Texel	53°03′96.71″N	4°71′22.77″E	nd	10.1	10.4
NL04	Netherlands	12/01/2016	Texel	53°03′97.68″N	4°71′24.59″E	nd	10.25	10.9
NL05	Netherlands	12/01/2016	Texel	53°03′97.68″N	4°71′24.59″E	nd	9.7	9.1
GER05	Germany	13/01/2016	Büsum	53°03′97.68″N	4°71′24.59″E	12	10.7	12.5
UK01	England	22/01/2016	Hunstanton	52°94′73.46″N	0°48′86.9″E	nd	13.8	28.0
UK02	England	24/01/2016	Gibraltar Point	53°09′40.11″N	0°33′72.98″E	nd	14.6	33.5
UK03	England	24/01/2016	Gibraltar Point	53°09′40.11″N	0°33′72.98″E	nd	14.7	34.5
UK04	England	24/01/2016	Skegness	53°13′99.82″N	0°34′96.33″E	nd	13.5	26.1
GER06	Germany	31/01/2016	Kaiser-Wilhelm-Koog	53°94′25.94″N	8°90′02.14″E	12	10.8	12.9
GER07	Germany	31/01/2016	Kaiser-Wilhelm-Koog	53°94′25.94″N	8°90′02.14″E	15	11.2	14.4
GER08	Germany	31/01/2016	Kaiser-Wilhelm-Koog	53°94′25.94″N	8°90′02.14″E	10	11.0	13.6
GER09	Germany	31/01/2016	Kaiser-Wilhelm-Koog	53°94′25.94″N	8°90′02.14″E	12	10.25	10.9
GER10	Germany	31/01/2016	Kaiser-Wilhelm-Koog	53°94′25.94″N	8°90′02.14″E	10	11.3	14.8
GER11	Germany	31/01/2016	Kaiser-Wilhelm-Koog	53°94′25.94″N	8°90′02.14″E	11	11.4	15.3
GER12	Germany	31/01/2016	Kaiser-Wilhelm-Koog	53°94′25.94″N	8°90′02.14″E	12	10.5	11.8
GER13	Germany	03/02/2016	Büsum	54°16′82.24″N	8°73′38.62″E	15	12.0	18.0 (18.0*)
GER14	Germany	03/02/2016	Büsum	54°13′36.07″N	8°65′44.62″E	11	11.4	15.3 (15.0*)
UK5	England	04/02/2016	Old Hunstanton	52°95′91.84″N	0°50′29.95″E	nd	13.6	26.8

The first cluster included the first two animals that were stranded on the small German archipelago Helgoland (GER 03&04), as well as the five sperm whales that stranded on the Dutch island of Texel (NL01-05) and finally the first animal that stranded on the British coast in Hunstanton (UK01). The second cluster comprises the group of seven sperm whales, which were found on February 1^st^ in the Kaiser-Wilhelm-Koog (GER06-12), followed by two individuals that stranded two days later near Büsum (GER13&14) and the individual that stranded the same day on February 3^rd^ at the British coast of Old Hunstanton (UK05). Two animals which stranded on January 8^th^ and 23^rd^ in Wangerooge (GER01) and Gibraltar Point (UK03) respectively, are also assigned with this second cluster. Four “solitaires” were observed which were not classifiable by this technique and included animals that stranded in January in Germany (08/01 Wangerooge GER02 & 13/01 Büsum GER05) and UK (Gibraltar Point UK02 & Skegness UK04 both on 23/01). The discrimination between the two stranding clusters is summarized in a cluster plot (Fig. 2b).

**Figure 3:**
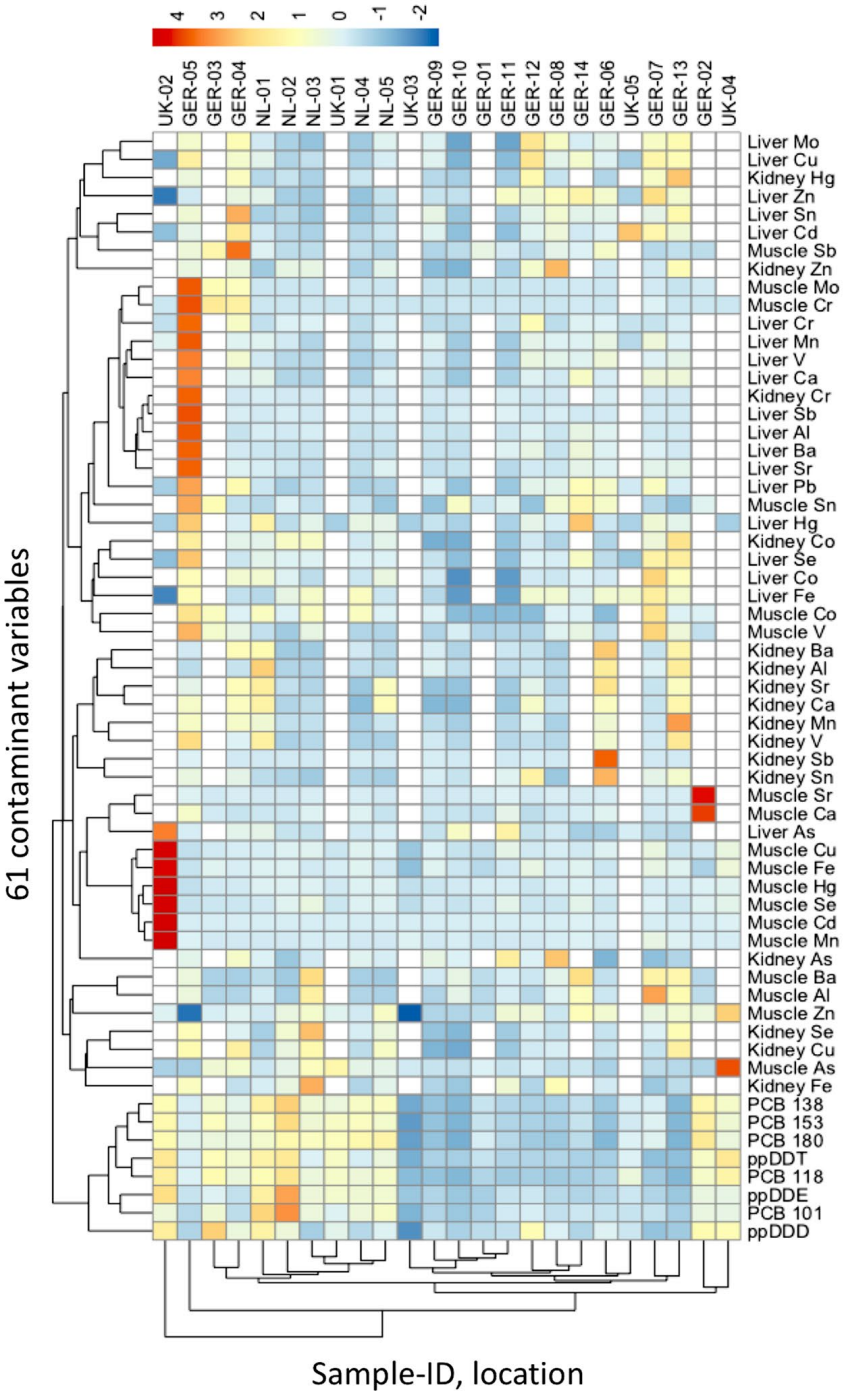
A heatmap based on 61 variables associated with organic compound and element concentrations in either muscle, liver, kidney or blubber for all 24 necropsied sperm whales. The map shows a pair-wise display of two dendrograms which were generated using unweighted pair-group method with arithmetic average algorithm (UPGMA). The individual assemblage dendrogram is on the y-axis and the assemblage of the organic compound and element concentrations in either muscle, liver, kidney or blubber dendrogram is on the x-axis. The spectrum of colours ranging from blue (low concentrations) to red (high concentrations).

The heatmap of the organic compound and element concentrations for muscle, liver, kidney and blubber of all 24 sperm whales shows a pair-wise display of two dendrograms, which were generated using unweighted pair-group method with arithmetic average algorithm (UPGMA) (Fig. 3). The spectrum of colours ranging from blue (low concentrations) to red (high concentrations) for the respective compounds gave two main patches of high concentration colours indicating the stranding cluster patterns. To conclude, the stranded sperm whales in the ‘January’ cluster showed higher concentrations of organic compounds compared to the individuals of the ‘February’ stranding cluster, which presented higher concentrations of elements in muscle, liver and kidney.

**Figure 4:**
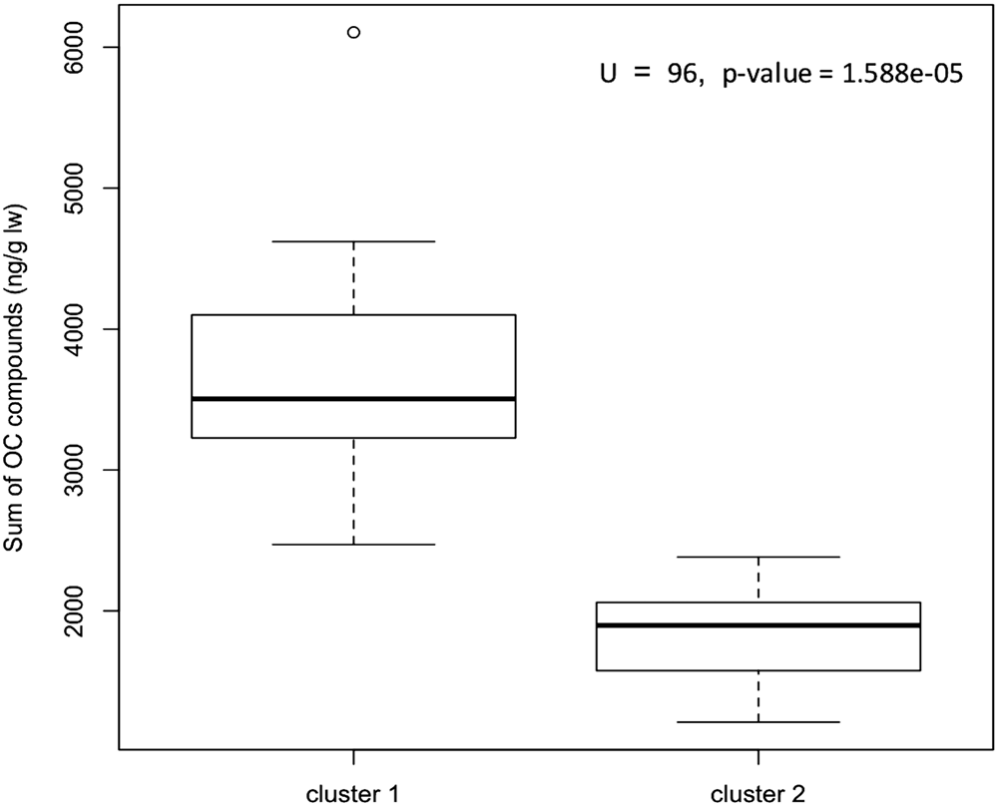
Boxplot of the sum of persistent organochlorine compounds measured in the blubber of sperm whale individuals from cluster 1 (January) & 2 (February).

**Figure 5:**
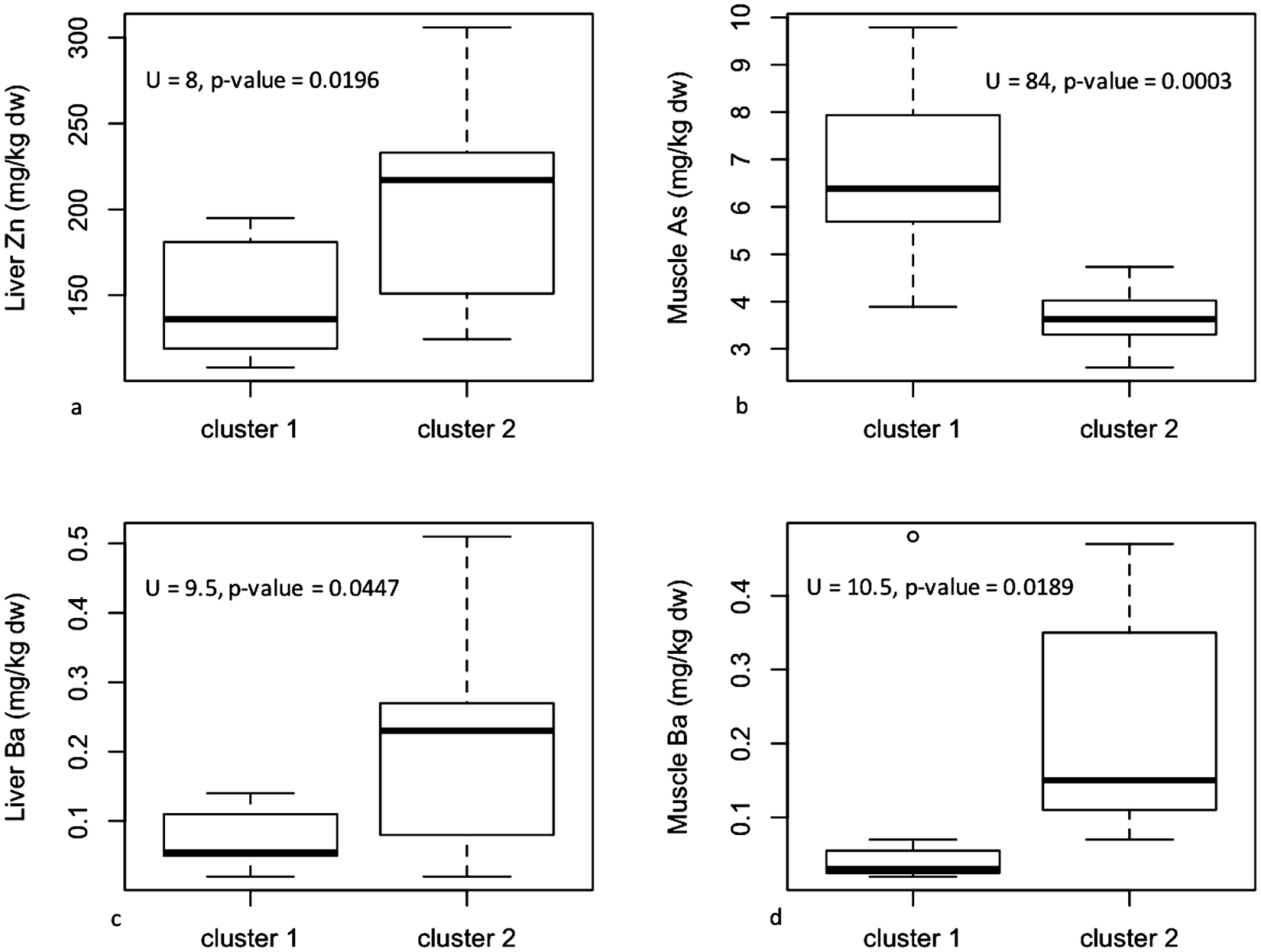
Boxplot of (**a**) liver zinc, (**b**) muscle arsenic and (**c**,**d**) liver and muscle barium concentrations measured in the sperm whale individuals from cluster 1 (January) & 2 (February).

To determine the most important variables that explains the clustering, we compared the means of concentrations of the 61 variables associated with organic compound and element concentrations in muscle, liver, kidney and blubber between the two largest clusters. The organic compound concentrations are more than twice as high in the individuals from the ‘January’ stranding cluster compared to the animals from the ‘February’ cluster (U = 96, p-value = 1.588e-05; Fig. 4). For the element concentrations, the main drivers of differentiation between the two stranding clusters were liver Zinc (Zn) concentrations (U = 8, p-value = 0.0196), muscle Arsenic (As) concentrations (U = 84, p-value = 0.0003) and liver and muscle Barium (Ba) concentrations (U = 9.5, p-value = 0.0447 and U = 10.5, p-value = 0.0189, respectively). The ‘January’ cluster showed lower Zn but higher As concentrations compared to the ‘February’ cluster (Fig. 5a,b). The Ba concentrations are higher in individuals from the ‘February’ cluster for both liver and muscle (Fig. 5c,d).

In a genetic study including 27 individuals of this stranding event, no clear pattern of subgrouping was detected^28^. We utilized these results for the 24 whales of this study to investigate potential associations between genotype and the clusters defined by the contamination data (Table 2). With regards to their genetic composition, the clusters assign differently, when comparing them to various parts of the Atlantic Ocean^1^. The ‘January’ cluster exhibits mtDNA haplotype frequencies similar to those of the Central Atlantic/Canary Islands (CATL/CNI) but is significantly different from the North Atlantic (NA) and its partitions Western North Atlantic Ocean and North Sea (WNAO, NSEA). Contrary to this assignment, the ‘February’ cluster is significantly different from CNI but shows an affinity to the North Atlantic and its partitions (NA, WNAO, NSEA; Table 3). Both clusters differ significantly from the Gulf of Mexico (GMX) and Mediterranean Sea (MED) sperm whale populations.

**Table 2:** Diversity in mtDNA of stranded male sperm whales, sorted in cluster 1 (January) & 2 (February) based on contamination data.

	Haplotype diversity H	Nucleotide diversity (%)	Number of individuals	Haplotype frequencies			
				A	B	C	N
Cluster 1	0.750 ± 0.096	0.394 ± 0.301	8	2	0	3	3
Cluster 2	0.666 ± 0.091	0.271 ± 0.218	12	6	2	4	0
Other	0.833 ± 0.222	0.263 ± 0.260	4	1	2	0	1

Haplotype diversity H and nucleotide diversity are shown with standard deviation. Haplotype frequencies are given in total numbers of individuals.  “Cluster1”, “Cluster2” and “other” (UK04, GER05, UK02, GER03) (Autenrieth et al. 2018).

**Table 3:** Pairwise genetic difference (mtDNA) between stranded male sperm whales, sorted in cluster 1 (January) & 2 (February) based on contamination data, and Atlantic populations of known origin (Fixation index FST and p-value for the exact test of population differentiation.

	FST		Exact-test p value	
	Cluster1	Cluster2	Cluster1	Cluster2
CATL	−0.016	0.004	0.571	0.381
CNI	−0.085	0.141*	0.851	0.015*
GMX	0.509***	0.538***	<0.001***	<0.001***
MED	0.716***	0.751***	<0.001***	<0.001***
NA	0.043	−0.043	0.076(*)	0.958
NSEA	0.061	−0.058	0.059(*)	0.784
WNAO	0.081	−0.025	0.015*	0.628

Significance was evaluated by permutation tests (***p < 0.001; *p < 0.05; (*)p < 0.1; values with p < 0.05 are printed in bold).

Abbreviation: “NA” = Northern North Atlantic; “NSEA” = Northern North Sea; “WNAO” = Western North Atlantic; “CNI” = Canary Islands; “CATL” = central Atlantic; “GMX” = Gulf of Mexico; “MED” = Mediterranean Sea (Alexander *et al*.^1^, Supplementary Material 2, and references therein).

## Discussion

Whereas female sperm whales are indisputably social, the situation is not as clear among males^9^. When separated from females, males may be found in aggregations covering a few kilometres or more^2^, but it is not clear if they are actually social. Previous observations came from the time of whaling and social structures within bachelor groups have only been described informally. These studies inconstantly reported loose associations of young males off California, scattered over large areas^29^, whereas tight pods of males were reported from the Azores and New Zealand^30,31^. It was concluded that large bachelor groups of males frequently split up and rejoin over a large area^2^, but the fidelity of their membership was doubtful. More recent research on sperm whales is equivocal, with some studies illustrating little apparent characteristics of social structure of males^6,10,11,32^, while another recent study even showed that immature males form long-term relationships^33^. A recent study on size and shape variations of the bony components of sperm whale cochleae suggested already that individuals might be affiliated to different bachelor pods^34^. The present study assessed whether variations of the contamination profile are indicative of spatial aggregation (and putatively social structures) of young male sperm whales as they might reflect a variation in feeding preferences and/or in feeding grounds as diet is the main relevant exposure pathway for marine mammals^12^. Ecological, biological and physiological factors that may lead to different contamination signatures in individual marine mammals were considered. As all stranded sperm whales were males, we could exclude sex-related differences in metabolism. Any differences in pattern could however occur due to the transfer of pollutants during pregnancy and lactation^35,36^ which would be influenced by the geographical origin and relative contaminant burden of their mother. Bioaccumulative pollutants are known to correlate positively with age, but our individuals formed a rather homogenous group according to their size and age. The observed variations in tissue concentrations and patterns of organic compounds and trace elements among different individuals of the stranded sperm whales allowed us to classify these animals in two main clusters. A first insight into the biological relevance of these clusters is provided by the coherence of the chronology of the stranding events.

### Chronological sequence of strandings

This discussion is limited exclusively to the individuals that have been necropsied, thus not all stranded animals are included, for a complete overview of the strandings we refer to an extensive study on the largest recorded sperm whale mortality event in the North Sea region presented elsewhere^27^. On January 8^th^, 2016 two dead sperm whales were found on the German Wadden Sea island Wangerooge. These animals were not associated within one of the cluster and therefore probably formed a group of spur-of-the-moment consociates of two solitary traveling sperm whales or joined one of the groups lately, sharing only the very last time their spatial aggregation. Four days later, on January 12^th^, more dead sperm whales were found in Germany, with two drifting carcasses near the island of Helgoland. That same afternoon, five sperm whales stranded on the Dutch island of Texel. These animals were grouped in one ‘January’ cluster that probably represent a bachelor pod composed of persistent companions that might have grouped together for a longer time period and shared a longer life history showing similarities in prey preferences and/or in location of feeding grounds. In Germany, on January 13^th^ another sperm whale stranded near Büsum, which was classified as solitary based on a different contamination pattern compared to the others.

The event continued in Hunstanton, England, where on January 22^nd^ a small group of sperm whales was observed in the shallows. One of these animals live stranded on rocks close to shore and died that night, while three other sperm whales, presumed to be the same individuals as those seen alive earlier, were found dead stranded nearby over the next days: on January 24^th^ two sperm whales were found at Gibraltar Point and one at Skegness. According to our analysis, these animals did not seem to belong to a bachelor pod formed by constant companions. These individuals were either classified as solitary or associated to one of the two clusters. It is more probable that they belonged to a group of spontaneous association that did not share a long-life history, showing no clear common pattern in their contamination profile. However, it must also be said that the dataset of these animals was not so powerful, since these individuals were not completely dissected and no liver and kidney samples were available.

On January 31^st^, seven sperm whales were found stranded in the Kaiser-Wilhelm-Koog, Dithmarschen, Germany, with two still alive at the time of stranding. On February 3^rd^, two dead sperm whales were found near Büsum, Germany. Finally, on February 4^th^, one sperm whale live stranded at New Hunstanton, England, where it died the same day. These animals are all assigned to one ‘February’ cluster and formed probably a bachelor pod of constant companions.

### What differentiates these groups?

The similarities in contamination profiles indicate that these individuals shared a significant part of their life history, showing concordances in prey preferences and/or in feeding grounds. The POP and TE patterns supported the social segregation of sperm whales in two separate bachelor groups for a longer time period. Higher PCB and DDT concentrations were observed in the ‘January’ stranding cluster that regrouped individuals that stranded on the German and Dutch coast. It is likely that the habitat where these animals feed strongly influences their POP profiles^12–16^. Higher pollutant burdens are generally found in species inhabiting coastal regions due to the close proximity of these animals to possible emissions, discharges and losses of POPs in temperate areas^20,21,25^. Thus, the animals of the ‘January’ cluster foraged in more polluted areas, probably more southern regions. This ascertainment is also supported by the fact that the individuals of the ‘January’ cluster show higher levels of muscular As concentrations. The environmental presence of As derives from both natural and anthropogenic sources^26^. Many natural processes contribute to environmental background concentrations of As, including pedogenesis, dust storms, volcanic eruptions, geothermal/hydrothermal activity, and forest fires, but As is a ubiquitous component of active and fossil geothermal systems such as the intraplate at the Azores and volcanic hotspots at the Canary Islands and Cape Verde^37^.

The individuals of the ‘February’ cluster on the other hand showed lower POP and As levels and higher Zn and Ba levels. The dissolved Zn profile in the ocean is nutrient-like, with near-zero concentrations in surface waters and maximum concentrations below 1000 m depth^38^. Dissolved Ba is a quasi-conservative tracer of Arctic water masses^39^. To conclude, these observations support the idea that the individuals of the ‘February’ cluster foraged in deeper North Atlantic feeding grounds around the Norwegian shelf edge.

### Genetic relationship and kinship

With regard to a putative geographic origin of the two clusters identified by contaminant analysis, the frequencies of mitochondrial control region haplotypes appear highly informative: Haplotype “N” occurred in the ‘January’ cluster in 3 out of 8 specimens (frequency = 37.5%). This is a statistically significant overrepresentation under the assumption that ‘January’ cluster specimens originate from the Northern part of the Atlantic Ocean, where this haplotype is very rare (frequency 1.1–5.5%). However, the ‘January’ cluster almost exactly resembles the haplotype N frequency of the Canary Islands (35.7%^28^). Unless one assumes that three brothers stayed together after birth, which is unlikely in light of known social behaviour and age of the stranded specimens, ‘January’ cluster haplotype frequencies indicate that these specimens came from the Central Atlantic, most likely the area of the Canary Islands. This is further supported by the significant differentiation the ‘January’ cluster shows against populations of the North Atlantic Ocean. Contrary to this, the second cluster shows significant genetic differences to the Canary Islands, while there is no genetic differentiation of the ‘February’ cluster against the North Atlantic regions (NA, NSEA, WNAO^1^). In summary, combining contaminant and genetic data, there is evidence for at least two cohorts of different origin among the stranded sperm whales, one from the Canary Islands and one from the northern part of the Atlantic.

### What does this mean for group cohesion?

Groups or social associations are not always easily identifiable through stranding data. Bachelor groups of sperm whales have previously been described as rather loose associations of immature males, which apparently communicate over considerable distances and which may join and split up again^4^. These apparently unstructured associations showed exceptionally rarely socializing behaviour at the surface^10,40^ and if, then for no more than a couple of hours^41^. Such loose association fits with the group of sperm whales that stranded on the English coasts, as they did not show a common contamination profile. However, witness reports indicated that the social cohesion within such male associations prevent animals from stranding or that they do not leave stranded individuals^3,4,42^. A recent study showed that immature males form long-term relationships occurring in tight surface groups that exhibit all types of surface behaviour seen within social units^33^. Our results from the ‘January’ and ‘February’ clusters imply likewise that sexually immature individuals in bachelor groups can form long-term associations. Young males may benefit in several ways from belonging to stable all-male groups, e.g., by feeding cooperatively and sharing information on the location of prey patches or by experiencing a reduced predation risk. Group living may also allow individuals to establish breeding alliances and practicing fighting skills with other males and cooperative behaviour against other males^43^. There is some evidence that these group living males are not necessarily related to each other, or share group-specific feeding specializations^41^.

According to our analysis, the remaining four individuals were solitary males. Usually, sperm whales become solitary upon nearing sexual maturity at around 27 years of age and lengths of approximately 13.7–15.2 meters^2^. These large bulls are more frequent in polar waters near higher latitudes^2^. The four ‘solitaires’ of our study were not the largest individuals (9.6–12 meters) but showed individual contamination profiles indicating that they might be either real solitary young individuals or distant members of a loose group.

### Explanation approaches for multiple strandings

Sperm whale strandings have been documented in the North Sea since the end of the 16^th^ century^42^ and occurred mostly in winter months between November and February in the period of male southward migration. All documented individuals were young males with a body length between 12 to 18 m^42^. The very shallow North Sea with a local coastline characterized by an intricate system of sand banks, mudflats, sandy islands and estuaries may have become a death trap because it is totally unsuitable for these deep-diving oceanic animals. Many theories have attempted to explain the phenomenon of sperm whale mass strandings, which probably result from complex interactions of physical (*e*.*g*. ocean currents, tides, geomagnetic anomalies, positive temperature anomalies and coastal configuration), biological factors (*e*.*g*. social behaviour, food availability, echolocation or orientation failure and diseases)^44–50^ and potentially more recently, anthropogenic factors such as marine noise. A recent paper suggested that solar storms may have triggered this sperm whale strandings^51^. Our study does not explore stranding causes but indicates that at least two separate groups of sperm whales made the same mistake to enter into the North Sea, which could be indicative of a common cause or influence which has mislead the sperm whales.

## Conclusions

The results of the study revealed that the contamination profiles can be indicative of social structures for young male sperm whales. The assessment which contaminants discriminated efficiently between individuals highlighted geographical indicator elements that have the potential to be used as ecological tracers. This study highlights the importance of combining different types of data. Whilst genetic data unravels genetic relationship and kinship, contamination data integrates areas, where animals occur during their lifetime. Especially in long lived animals with a large migratory potential, such as sperm whales, contamination data may carry highly relevant information about aggregation through time and space. With the addition of genetic data, such spatiotemporal aggregations (i.e., clusters in the contaminant analysis) can be assigned to their putative origin.

## Materials and Methods

### Specimens

Multiple sperm whale stranding events occurred at different locations around the North Sea during January and February 2016. During this period, thirty dead animals were observed along European coasts. Twenty-four animals were necropsied and sampled after their discovery (Table 1). Age determination of sperm whales was realized by counting growth layer groups (GLG’s) in the teeth^43^.

### Determination of trace elements and persistent organic pollutants

Two different laboratories conducted chemical determination of trace elements and persistent organic pollutants in sperm whale samples. The Laboratory of Animal Ecology and Ecotoxicology (CART-LEAE, ULiège), in Liège, Belgium conducted the analysis of the animals stranded on the Dutch and German coast (NL-01 → NL-05 and GER-01 → GER-14). Their methods for sample analysis of organochlorines and metals are as described in Schnitzler *et al*. and Pinzone *et al*.^16,52^. The Centre for Environment, Fisheries and Aquaculture Science (Cefas) in Lowestoft, UK of the animals stranded on the UK coast (UK-01 → UK-05). Their methods for sample analysis of organochlorines and metals are as described in Al-Zaidan *et al*.^53^. A detailed description can be found in the Supplementary Materials.

### Genetic relationship and kinship

To put the genetic results from Autenrieth *et al*. (2018) in perspective with the contamination data we utilized the genetic data of the 20 samples used in this study, which were assigned to two clusters based on contamination data^28^. We compared mitochondrial haplotype frequencies within these clusters to the overall Atlantic subpopulation data^1^. We computed pairwise fixation indices F_ST_, haplotype and molecular diversity using the program Arlequin3.5^54^. The exact test (no. of steps in Markov chain = 100,000; No. of dememorization steps: 10,000; significance level = 0.05) was then used to test for haplotype frequency differences between the two clusters and in comparison, to the other Atlantic subpopulations. For the AMOVA analysis, five regions (the Gulf of Mexico (GMX), Mediterranean Sea (MED), North Atlantic (NA), North Sea (NSEA), Western North Atlantic Ocean (WNAO)) for the Atlantic (ATL) and the two clusters were analysed.

### Statistical tests

The datasets generated and analysed within the current study are available from the corresponding author on reasonable request. All data are presented as means ± standard deviation. To determine toxicological profile similarities among individuals, a matrix of pairwise Euclidean distances was calculated from the means of the measured concentrations, and a hierarchical cluster analysis based on this matrix was performed using the unweighted pair-group method with arithmetic average algorithm (UPGMA) (package ‘cluster’^55^). The ‘NbClust’ package was used to determine the optimal number of clusters in a data set and to choose the best clustering scheme^56^. The cophenetic correlation coefficient was computed to indicate the degree to which distances in the resulting dendrogram accurately represent the original inter-individual distances^57,58^. The ‘factoextra’ package allowed the extraction and visualization of the results of these multivariate data analyses^59^. The visualization of the contamination profile in a graphical format, in form of a heatmap helped with the understanding and interpretation (package ‘pheatmap’^60^). To determine the most important variables that permit the clustering, we compared the medians between the two largest clusters. All the analyses were performed in R 3.1.1^61^. Statistical significance was accepted at p < 0.05.

## Electronic supplementary material

Supplementary Materials
