# Supplementary material for: Inter-individual differences in contamination profiles as tracer of social group association in stranded sperm whales
Source: Sci Rep. 2018 Jul 19;8:10958. doi: 10.1038/s41598-018-29186-z (PMC6053436; doi:10.1038/s41598-018-29186-z)
Supplement: Supplementary file 1 — Supplementary Materials [file 41598_2018_29186_MOESM1_ESM.pdf]

Inter-individual differences in contamination profiles as tracer of social group association in stranded sperm whales

Joseph G. Schnitzler[1*], Marianna Pinzone[2], Marijke Autenrieth[3], Abbo van Neer[1], Lonneke L. IJsseldijk[4], Jonathan L. Barber[5], Rob Deaville[6], Paul Jepson[6], Andrew Brownlow[7], Tobias Schaffeld[1], Jean-Pierre Thomé[8], Ralph Tiedemann[3], Krishna Das[2], Ursula Siebert[1]

[1] Institute for Terrestrial and Aquatic Wildlife Research, University of Veterinary Medicine Hannover, Foundation, 25761 Büsum, Schleswig-Holstein, Germany.

[2] Freshwater and Oceanic sciences Unit of reSearch - Oceanology, University of Liège, Allée du 6 Août, B6C, 4000 Liège.

[3] Unit of Evolutionary Biology/Systematic Zoology, Institute for Biochemistry and Biology, University of Potsdam, Karl-Liebknecht-Str. 24-25, 14476 Potsdam, Germany

[4] Faculty of Veterinary Medicine, Department of Pathobiology, Utrecht University, Yalelaan 1, 3584CL Utrecht, The Netherlands

[5] Centre for the Environment, Fisheries and Aquaculture Science (Cefas) Lowestoft Laboratory, Pakefield Road, Lowestoft, Suffolk, NR33 0HT, UK

[6] CSIP, Institute of Zoology, Regent's Park, London, NW1 4RY, UK

[7] SRUC Wildlife Unit, Drummondhill, Inverness, IV2 4JZ, UK

[8] Laboratory of Animal Ecology and Ecotoxicology (CART-LEAE) B6c, Liège University, Liège, Belgium

[*]Corresponding author

Email addresses:

JS:	joseph.schnitzler@tiho-hannover.de	Tel: +49 511-856 8156
MP:	mpinzone@ulg.ac.be	
MA:	marauten@uni-potsdam.de	
AVN:	Abbo.van.Neer@tiho-hannover.de	
LIJ:	L.L.IJsseldijk@uu.nl	
JB:	jon.barber@cefas.co.uk	
RD:	Rob.Deaville@ioz.ac.uk	
PJ:	Paul.Jepson@ioz.ac.uk	
AB:	andrew.brownlow@sruc.ac.uk	
TS:	Tobias.Schaffeld@tiho-hannover.de	
JPT:	jp.thome@ulg.ac.be	
RT:	tiedeman@uni-potsdam.de	
KD:	krishna.das@ulg.ac.be	
US:	Ursula.siebert@tiho-hannover.de	

Supplementary Materials

Two different laboratories conducted chemical determination of trace elements and persistent organic pollutants in sperm whale samples. The Laboratory of Animal Ecology and Ecotoxicology (CART-LEAE, ULiège), in Liège, Belgium conducted the analysis of the animals stranded on the Dutch and German coast (NL-01→NL-05 and GER-01→GER-14). The Centre for Environment, Fisheries and Aquaculture Science (Cefas) in Lowestoft, UK of the animals stranded on the UK coast (UK-01→UK-05). A detailed description can be found here.

ULiege data

Samples preparation

Between 3.2 and 7.5g of muscle, liver and kidney were inserted separately in plastic vials, whose weight was previously measured, and homogenised with a ceramic knife and pence. Then, samples were freeze-dried for 48 hours (Martin Christ freeze dryer, Alpha 2-4 LDplus, 37520, Germany). Once dry, each vial was measured again to estimate the dry weight. Samples of blubber were cut vertically from the skin, trying to get the maximum amount possible of all blubber layers. In total, 19 samples of muscle, 17 of liver, 15 of kidney and 19 of blubber were used for contamination analyses.

Total mercury analysis

Between 0.003 and 0.02g of muscle, 0.0016 – 0.0025g of kidney and circa 0.001g for liver were weighted (0.001g precision) and inserted in quartz boats. Total mercury (T-Hg) was measured via atomic absorption spectroscopy (AAS, Direct Mercury Analyser DMA-80, Milestone Inc., Shelton, USA). Quality control was assured by measuring blanks (HCl 1%) levels and standardized solution (0.1mg Hg L^{-1} and 1mg Hg L^{-1}) before and after every analysis. Finally,

the drift of the instrument was measured using Certified Reference Material at the beginning and at the end of each analysis series: DORM-2 (dogfish muscle: 4.64 mg Hg kg^{-1}) and DOLT-3 (dogfish liver: 3.37mg Hg kg^{-1}).

Trace element analysis

Between 0.09 and 0.11g for liver and kidney, between 0.19 and 0.21g for muscle were weighted and inserted in Teflon digestion vessels. Samples were mineralized for 25-30 minutes in a closed microwave digestion lab-station (Ethos D, Milestone Inc., Shelton, USA), using 2mL of nitric acid (HNO_3), 1mL of hydrogen peroxide (H_2O_2) reagents and 5mL of milliQ water 2.0 Ω. In total 18 trace elements (TEs: Be, Al, V, Mn, Co, As, Se, Mo, Ag, Sn, Sb, Bi, Cr, Fe, Ni, Cu, Pb and Cd) were determined by Inductively Coupled Plasma Mass Spectrometry using Dynamic Reaction Cell technology (ICP-MS, ELAN DRC II, Perkin Elmer SCIEXTM, Waltham, USA). Analytical quality control was conducted using a series of 10 blanks after the measurement of each tissue series and four Certified Reference Materials (CRM): DOLT-3 dogfish liver, DORM-2 dogfish muscle, NIST 1566b oyster tissue and NIST 1577c bovine liver. For each TE the detection limit (DL) and quantification limit (LQ) were calculated depending on blanks values.

Persistent organic pollutants analysis

The analysis of POPs slightly modified, based on the method from Damseaux et al., 2017 [62]. The extraction of NDL-PCBs (28, 52, 101, 118, 138, 153 and 180) and organochlorine pesticides (p,p'-DDT, p,p'-DDD, p,p'-DDE, α-HCH, β-HCH, γ-HCH and HCB) was performed on 250mg wet weight of blubber with a Soxhlet extraction using of acetone: *n*-hexane 1:1 (*v:v*). Before the extraction, 50µL of a hexanic solution of Mirex (Dr. Ehrenstorfer®, Augsburg, Germany) was added to samples as a surrogate internal standard at

100 pg µL-1. The fat content was determined gravimetrically after solvent evaporation, through the use of a TurboVap LV concentration Evaporator workstation (Zymark TurboVap®LV, Charlotte, USA). The clean-up of the extracts was conducted using first H_2SO_4 98% and then Florisil solid phase enrichment (Supelco, Envi-Florisil, Bellefonte, PA). Five µL of nonane (C_9H_{20}) were added as a keeper to the purified extract. Each extract was evaporated under a gentle stream of nitrogen till just the keeper remains in the vial. The final extract was reconstituted with 45 µL of n-hexane and 500 µL of Mirex (100 pg µL^{-1} in hexane) as injection volume internal standard (Dr. Erhenstorfer® GmbH, Augsburg, Germany). This compound such as Mirex were never detected in such samples from these locations during pre-test analysis. Finally, these extracts were analysed by high-resolution gas chromatography (Thermo Quest Trace, 2000; Thermo Quest, Milan, Italy) equipped with a ^{63}Ni electron capture detector (ECD) and on column injector. Pollutants were analysed on a 60 m x 0.25 mm (0.25 mm film) DB5 ms capillary column (J&W Scientific, USA). Other analytical parameters were described elsewhere [63]. The quantification was performed by means of the internal standard method. A calibration curve (1.5 - 250 pg µL^{-1}) was established for each compound of interest. The confirmation of the identity and concentrations of the compounds of interest were periodically performed with a high resolution gas chromatograph coupled to an ion trap mass spectrometer (Trace GC Ultra and ITQ 1100 from ThermoQuest). The transfer line temperature was kept at 290°C and the ion trap temperature was set to 250°C. The electron ionization (EI) was performed at 70 eV and the ion trap was operating in MS/MS mode. The quality control (QC) was pork fat, free of the compounds of interest. The pork fat was spiked with nominal concentrations of NDL-PCBs and organochlorine pesticides of 5 ng g-1 lipid weight forming the QC. Recovery rates ranged between 75% and 109% for QC according to requirements of SANCO (SANCO, 2014). The limit of detection (LOD) was 0.02 ng g-1 lipid weight and the

measured limit of quantification (LOQ) determined with PCB spiked lard was measured at 0.7 ng g^{-1} lipid weight.

Cefas data

Sperm whale tissue preparation

Samples were stored frozen at -20°C until required for analysis. In preparation for analysis, blubber, muscle and liver samples were defrosted and dissected in a strictly controlled, contaminant-free environment. Sub-samples (~5g) of blubber (n=5) were processed for PCBs and OCPs, and sub-samples (~3g) of muscle (n=5) and liver (n=2) were processed for metals analysis. Tissue samples were stored frozen at -20°C until required for analysis.

Sperm whale muscle and liver metals analysis

Samples underwent an acid digestion using an enclosed vessel microwave (Milestone ETHOS UP, Analytix Ltd, Boldon, UK). Typically, approximately 3 g of homogenised sample was weighed out and pre-digested overnight in 6mL of nitric acid (Aristar grade 69%, VWR, Leicestershire, UK). The digestion was performed using a temperature-controlled microwave programme specific for the sample matrix. The digest was then further diluted prior to analysis by inductively-coupled plasma-mass spectrometry (ICP-MS), using an Agilent 7900 (Agilent Technologies, Waldbronn, Germany), with indium as the internal standard. Quantification of trace elements was performed by external calibration using 8 calibration levels (0-500ng/ml).

Sperm whale blubber extraction and drying for organochlorine analysis

After thawing, the homogenised subsamples were dried by mixing with anhydrous sodium sulphate and storing in a freezer for a minimum of 12 hours prior to further analysis. The samples were subjected to Soxhlet extraction using of acetone: n-hexane 1:1 (v:v) for 5.5 hours.

The total extractable lipid content was determined gravimetrically after evaporation of the solvent from an aliquot of the uncleaned extract. Depending on the lipid content of the samples, varying volumes of the biota extracts were cleaned to have ~50 mg of lipid in the samples for PCB and OCP analysis. If concentrated extracts contained water, they were further dried using a sodium sulphate column, eluted with hexane until 16 mL of eluate had been collected.

Clean-up and analysis for PCBs and OCPs analysis

An aliquot of the blubber extracts was cleaned up and fractionated using alumina (5% deactivated) and silica (3% deactivated) columns, respectively. The silica column fractionation results in two fractions, the first fraction containing polychlorinated biphenyls (PCBs), HCB and p,p'-DDE, the second fraction containing the remaining OCPs. The final GC-ready fractions were spiked with PCB53 internal standard and made up to a final volume of 1 ml. PCB and OCP concentrations in blubber extracts were determined with an Agilent 6890 GC with µECD. The separation of analytes was performed on a 50.0 m × 200 µm, 0.33-µm-film-thickness DB-5 capillary column (J&W). The carrier and ECD make-up gas were hydrogen (32.2 psi constant pressure, initial velocity 50 cm/s) and argon/methane (95:5), respectively. The initial oven temperature was 90°C, held for 2.00min, then increased to 165°C at 15°C/min, to 285°C at 2°C/min, and finally held for 23 min. The injector temperature and detector temperature was 270°C and 300°C, respectively. A 1-µl extract was injected in splitless mode with a purge time of 2 min. The PCB standard solutions contained the following 27 compounds in iso-octane: Hexachlorobenzene, p,p'-DDE, CB101, CB105, CB110, CB118, CB128, CB138, CB141, CB149, CB151, CB153, CB156, CB158, CB170, CB18, CB180, CB183, CB187, CB194, CB28, CB31, CB44, CB47, CB49, CB52 and CB66; together with the internal standard CB53. Quantitation was performed using internal standards and 7 calibration levels (range 0.5 – 100ng/ml). The OC standard solutions contained the following 5 compounds in

iso-octane: alpha-HCH, gamma-HCH, dieldrin, p,p'-TDE and p,p'-DDT; together with the internal standard CB53. Quantitation was performed using internal standards and 7 calibration levels (range 0.5 – 100ng/ml).

QA/QC

The laboratory biannually participates in proficiency testing scheme Quasimeme (Quality Assurance of Information for Marine Environmental Monitoring in Europe) as external quality assurance. All analyses were carried out under full analytical quality control procedures that included the analysis of certified reference material(s) and a blank sample with every batch samples analysed so that the day-to-day performance of the methods could be assessed. If levels of target analytes in the samples were outside of the range of the instrument calibration, extracts were diluted to be within range and re-analysed. Reference materials used were BCR349 (cod liver oil; European Bureau of Community reference), DOLT-5 (dogfish liver; National Research Council Canada, Halifax, Nova Scotia, Canada) and DORM-4 (fish protein; National Research Council Canada, Halifax, Nova Scotia, Canada). The results obtained for the reference materials were plotted as Shewhart quality control charts for each compound or trace element determined. The charts had previously been created by the repeated analysis of the above certified reference materials in the Cefas Lowestoft Laboratory using the North West Analytical Quality Analyst software™ (Northwest Analytical Inc., USA). Warning and control limits had been defined for the charts as 2σ and 3σ – 2x and 3x the standard deviation from the mean for each compound or trace element, respectively. The results obtained for all samples analysed were accepted as valid as the results for the certified reference materials were within the limits set by the control charts.
